# Mastication as a Stress-Coping Behavior

**DOI:** 10.1155/2015/876409

**Published:** 2015-05-18

**Authors:** Kin-ya Kubo, Mitsuo Iinuma, Huayue Chen

**Affiliations:** ^1^Seijoh University Graduate School of Health Care Studies, 2-172 Fukinodai, Tokai, Aichi 476-8588, Japan; ^2^Department of Pediatric Dentistry, Asahi University School of Dentistry, 1851 Hozumi, Mizuho, Gifu 501-0296, Japan; ^3^Department of Anatomy, Gifu University Graduate School of Medicine, 1-1 Yanagido, Gifu 501-1194, Japan

## Abstract

Exposure to chronic stress induces various physical and mental effects that may ultimately lead to disease. Stress-related disease has become a global health problem. Mastication (chewing) is an effective behavior for coping with stress, likely due to the alterations chewing causes in the activity of the hypothalamic-pituitary-adrenal axis and autonomic nervous system. Mastication under stressful conditions attenuates stress-induced increases in plasma corticosterone and catecholamines, as well as the expression of stress-related substances, such as neurotrophic factors and nitric oxide. Further, chewing reduces stress-induced changes in central nervous system morphology, especially in the hippocampus and hypothalamus. In rodents, chewing or biting on wooden sticks during exposure to various stressors reduces stress-induced gastric ulcer formation and attenuates spatial cognitive dysfunction, anxiety-like behavior, and bone loss. In humans, some studies demonstrate that chewing gum during exposure to stress decreases plasma and salivary cortisol levels and reduces mental stress, although other studies report no such effect. Here, we discuss the neuronal mechanisms that underline the interactions between masticatory function and stress-coping behaviors in animals and humans.

## 1. Introduction

Stress is a physiologic and psychologic response to environmental changes and noxious stimuli. Chronic stress negatively affects physical and mental health [1–3], ultimately leading to disease [4–8]. Stress-related diseases are prevalent worldwide. Stress activates the neuroendocrine system via the autonomic system and hypothalamic-pituitary-adrenal (HPA) axis, which leads to the release of corticosteroids and hormones [9, 10].

Chewing, swallowing, and speaking are important oral functions related to physical, mental, and social health [11–13]. In particular, masticatory ability influences nutritional status, overall health, and activities of daily living, especially in the elderly population [14, 15]. Chewing ability is frequently impaired in the elderly, and many older adults develop dental problems as a result of tooth loss, which compromises general health status and is an epidemiologic risk factor for Alzheimer's disease [16–20]. In animals, impairing mastication by removing teeth results in impaired spatial learning due to morphologic changes in the hippocampus [20]. Thus, chewing appears to have an important role in maintaining some aspects of cognitive function [20].

Chewing is also an effective stress-coping behavior. When exposed to an inescapable stressor, animals assume coping behaviors, such as chewing, that attenuate some elements of the stress response [21]. In humans, nail-biting, teeth-clenching, and biting on objects are considered outlets for emotional tension or stress. Animals provided the opportunity to chew or bite wooden sticks during immobilization or restraint stress exhibit decreases in stress-induced plasma corticosterone levels and attenuated HPA axis and autonomic nervous system responses to stress, which helps to prevent the stress-induced formation of gastric ulcers [4, 22–24], deficits in spatial learning ability [25, 26], and bone loss [27].

In humans, gum chewing is reported to relieve stress and improve task performance [28–30]. A recent functional magnetic resonance imaging study revealed that gum chewing during exposure to a loud noise inhibits the propagation of stress-related information in the brain [31]. Data regarding the stress-attenuating benefits of gum chewing, however, are conflicting and difficult to replicate [32–34]. Here, we provide an overview of the mechanisms that underlie chewing as a stress-coping behavior in animals and humans.

## 2. Effects of Stress and Mastication

Mastication under stressful conditions prevents stress-induced ulcer formation in the stomach [4, 22–24], spatial cognitive deficits [25, 26], anxiety-like behavior [35], and osteoporosis [27]. Onishi et al. [36] reported that maternal chewing during prenatal stress prevents prenatal stress-induced learning deficits in the adult offspring. Several studies have demonstrated that chewing attenuates stress-induced functional and morphologic changes in the hippocampus [25, 36–40].

Spatial cognitive function is mainly controlled by the hippocampus. The hippocampus is sensitive to stress, as well as the aging process, and it is one of the first brain regions to be structurally and functionally modified by stress [41]. Stress-induced increases in corticosterone impair hippocampal-dependent learning and memory [42–44]. Recent reports indicate that chewing ameliorates stress-induced deficits in hippocampal-dependent spatial cognitive function. For example, Miyake et al. [25] reported that rodents given wooden sticks to chew on during immobilization stress exhibit attenuated stress-induced suppression of spatial memory and glucocorticoid receptor expression in the hippocampus. Chronic stress leads to the downregulation of corticosterone receptors and the inhibition of negative feedback from the hippocampus to the HPA axis [37]. Also in rats, active chewing during immobilization stress ameliorates the stress-induced impairment of N-methyl-D-aspartate receptor-mediated long-term potentiation [38], which may be due to chewing-induced activation of histamine H1 receptors [39]. In addition, aggressive mastication during stress prevents the stress-induced decrease in brain-derived neurotrophic factor mRNA and neurotrophin-3 mRNA in the hippocampus. Brain-derived neurotrophic factor plays an important role in long-term potentiation [45], neurogenesis [46], dendritogenesis [47], and activity-dependent neuroplasticity [48], consistent with the finding that chewing during stressful conditions ameliorates the stress-induced suppression of cell proliferation in the hippocampal dentate gyrus [40]. Cell proliferation in the hippocampal dentate gyrus strongly correlates with learning ability [49], and neurotrophin-3 influences the development of the hippocampus [50]. Nitric oxide levels are increased by restraint stress and, in rodents, biting suppresses the increases in the nitric oxide levels in the hypothalamus [51]. Analysis of blood flow in the amygdala and hypothalamus using laser Doppler flowmetry and O_2_-selective electrodes in rats allowed to chew on sticks under restraint stress revealed recovery of stress-induced decreases in PO_2_ levels [52]. Chewing may reduce nitric oxide by increasing PO_2_ levels in the hypothalamus, thus altering hemoglobin-scavenging activity for nitric oxide. Previous animal studies indicated that the stress-coping effects of chewing are mediated by the autonomic nervous system and the HPA axis.

Additionally, exposure to stress is a precipitating factor for many illnesses, including mood disorders [53]. In humans, dysregulation of thyroid hormones [54] and glucocorticoids [55] has long been associated with mood disorders. Helmreich et al. [35] reported that chewing on a wooden dowel during tail-shock in rats prevented stress-induced anxiety-like behavior and attenuated stress-induced decreases in thyroid hormone. The effects of chewing on thyroid and glucocorticoid levels may account for the effects of chewing to reduce stress-induced anxiety.

Osteoporosis is a skeletal disease characterized by low bone mass and microstructural bone deterioration, with an increased risk of fracture [56]. A large body of evidence from animal and human studies indicates a link between chronic mild stress and bone loss [57–59]. We examined the effects of chewing during chronic stress on stress-induced bone loss. Chewing under chronic stress prevents the increase in plasma corticosterone and noradrenaline levels, which attenuates both the reduced bone formation and increased bone resorption, and improves the trabecular bone loss and microstructural bone deterioration induced by chronic mild stress [27].

Prenatal stress increases the risk for neurobiological and behavioral disturbances in adult offspring [60, 61]. Clinical studies demonstrated that pregnant mothers exposed to social, emotional, or hostile experiences have offspring with an increased susceptibility as adults to mental disorders, such as depression, schizophrenia, and cognitive deficits [62]. We examined whether allowing pregnant mice to chew on a wooden stick during stress decreased the stress-induced learning deficits of the adult offspring by measuring plasma corticosterone levels, spatial learning ability, and cell proliferation in the hippocampal dentate gyrus of the adult offspring [36]. Allowing mouse dams to chew on a wooden stick during exposure to prenatal stress attenuated the increase in prenatal stress-induced plasma corticosterone levels. Further, adult offspring of dams exposed to prenatal stress exhibited impaired learning and decreased cell proliferation in the dentate gyrus, which was attenuated by allowing the dams to chew on a wooden stick during exposure to prenatal stress. Maternal chewing during prenatal stress thus appears to be effective for preventing learning deficits in the adult offspring [36].

### 2.1. Mastication and the Autonomic Nervous System

Mastication during stressful conditions suppresses stress-induced activation of the autonomic nervous system, causing sympathetic nerve terminals to locally release catecholamines [4, 22, 63]. Aggressive biting during exposure to stress significantly attenuates stress-induced increases in dopamine metabolism [64] and noradrenaline turnover in the hypothalamus and limbic areas [4, 63]. Okada et al. [65] reported that restraint stress-induced increases in blood pressure and core temperature were significantly suppressed in rats allowed to chew on a stick compared with rats that were restrained but not given a stick to chew, consistent with other reports [66]. In addition, chewing on a wooden stick during immobilization stress prevents poststress arrhythmias [67]. Interleukin- (IL-) 1*α*, IL-1*β*, and IL-6 have important roles in the thermoregulatory system [68] and allostasis [69]. IL-1*β* acts on the hypothalamus to enhance the secretion of corticotropin-releasing hormone [70, 71]. Stress induced by placing an animal in an open-field box leads to increased plasma IL-6 levels [72]. Therefore, biting induced inhibition of the stress-related increase in the core temperature might be due to the suppression of serum IL-1*β* and IL-6 levels. Cytokines such as IL-1*α*, IL-1*β*, and IL-6 are also involved in immunity. Some studies indicate that mastication-induced suppression of these cytokines prevents gastric ulcer formation [23, 24, 63, 64]. An animal study using micro-positron-emission tomography showed that chewing during immobilization stress suppresses the stress-induced increase in plasma corticosterone levels and glucose uptake in the paraventricular hypothalamic nucleus and anterior hypothalamic area, but not in the lateral hypothalamus [73].

### 2.2. Mastication and the HPA Axis

Mastication during stressful conditions suppresses activation of the HPA axis. In rats and mice, chewing or biting on wooden sticks under various stressors such as immobilization, restraint, cold exposure, and tail pinch attenuates the secretion of adrenocorticotropic hormone (ACTH) [38, 74, 75] and plasma corticosterone levels [23, 35, 38, 40, 63, 74, 76, 77]. Suppression of ACTH secretion may account for subsequent changes in physiologic stress markers in the paraventricular nucleus of the hypothalamus. Mastication under stress-inducing conditions suppresses the stress-activated expression of corticotropin-releasing factor, which controls ACTH secretion [78]; c-Fos, an indirect marker of neuron activity [79]; the phosphorylation of extracellular signal-regulated kinases 1/2 [80]; and the expression of nitric oxide synthase mRNA [81] and levels of nitric oxide [82], which is an important signaling molecule in corticotropin-releasing factor release [83] in the paraventricular nucleus of the hypothalamus. The negative feedback mechanism of the HPA axis reduces the secretion of glucocorticoids mainly by inhibiting the hypothalamic and hypophyseal activities and indirectly by binding to glucocorticoid receptors in the hippocampus [84]. Chewing ameliorates the stress-induced downregulation of glucocorticoid receptors, which suppresses negative feedback mechanisms [25]. In addition, biting on a wooden stick during chronic stress decreases neuronal nitric oxide synthase mRNA expression in the hypothalamus [81], which may be involved in the regulation of corticotropin-releasing factor secretion. Koizumi et al. [67] reported that chewing during immobilization stress prevents poststress arrhythmias in rats. Cardiovascular activity is controlled by the hypothalamus [85]. These effects of chewing on the HPA axis also ameliorate stress-induced cardiac imbalances and reduce susceptibility to stress-induced arrhythmias by inhibiting neuronal responses in the hypothalamus.

### 2.3. Neuronal Mechanisms That Underlie Stress Attenuation by Chewing

How does chewing during stress-inducing conditions suppress the autonomic nervous system and HPA axis? We suggest that stress-coping activities such as chewing engage the medial prefrontal cortex (mPFC) and the right central nucleus of amygdala neuronal activity asymmetrically [86]. The mPFC is critically involved in the regulation of stress-induced physiologic and behavioral responses [87–90]. Dopamine mainly controls the stress-related actions of the mPFC [91, 92]. Mice and rats exposed to an inescapable stress will chew on an inedible material, such as aluminum foil or cardboard, in the cage [76, 93]. Under inescapable stress conditions, chewing suppresses increases in plasma corticosterone [94]. Moreover, chewing also attenuates stress-related dopamine utilization preferentially within the mPFC [93]. Chewing-induced suppression of mPFC dopamine utilization is largely confined to the right hemisphere [93]. Together, these observations suggest a particularly important role for the right mPFC in stress-coping behavior. Chewing leads to an increase in fos-immunoreactivity that is selective for the right mPFC and a decrease in fos-immunoreactivity that is selective for the central nucleus of the right amygdala, a region that may regulate dopamine, both of which are implicated in regulating dopamine utilization in the mPFC, particularly under stress-inducing conditions [94–96]. In addition, chewing during stress-inducing conditions also attenuates the stress-induced release of noradrenaline in the amygdala [4, 22, 63]. Therefore chewing-induced changes in catecholamines in the mPFC and right central nucleus of the amygdala play an important role in stress-coping behavior.

A possible mechanism for chewing-induced alterations in hippocampus-related behavioral and morphologic changes is the brain histaminergic reaction. Chewing induces histamine H1 release in the hippocampus, and H1 receptor activation might recover stress-suppressed synaptic plasticity. The mesencephalic trigeminal nucleus receives proprioceptive sensory inputs via the trigeminal nerve from the masseter muscle spindle and the periodontal ligaments during mastication [97]. A subpopulation of the mesencephalic trigeminal nucleus neurons projects its fibers into the tuberomammillary nuclei (TMN) of the posterior hypothalamus in which histaminergic neuronal cell bodies are localized [98, 99]. Chewing increases the hypothalamic histamine concentration, thereby increasing satiety [100, 101], suggesting that chewing stimulates histaminergic neurons in the TMN. Axons of histaminergic neurons in the TMN project widely throughout the entire brain, including the hippocampus [102–105], and electrical stimulation of the TMN facilitates extracellular concentrations of histamine in the hippocampus [106]. Thus, a chewing-induced increase in the histamine level in the hippocampus might rescue long-term potentiation via the recovery of stress-attenuated N-methyl-D-aspartate receptors [39].

## 3. Human Studies

### 3.1. Sleep Bruxism and Stress

Sleep bruxism is a stereotypic movement disorder that is characterized by grinding or clenching the teeth during sleep and is generally associated with sleep arousal [107]. Sleep bruxism results in damage to the teeth, periodontal tissues, and masticatory muscles, as well as cervical pain and temporomandibular disorders [108]. The onset of sleep bruxism peaks between 20 and 45 years of age, although it also occurs in children [107, 109, 110]. Sleep bruxism is common in females [111]. Although the complete etiology of sleep bruxism is not clear, some factors include occlusal interference [112], psychosocial stress [113–115], psychologic stress [113, 114, 116–118], smoking [113], striatal D2 receptor activation [119], and transient sleep arousal [120]. Some studies suggest that stress is a causal agent of sleep bruxism because sleep bruxism occurs more often after exhausting and stressful days [121]. In an epidemiologic study on British, German, and Italian populations, self-reported sleep bruxism was positively related to a highly stressful lifestyle [116] and significantly associated with severe stress at work [117]. An analysis of stress-coping strategies in patients with sleep bruxism compared to nonbruxing controls indicated that sleep bruxism patients utilize significantly fewer positive coping strategies such as escape [118, 122]. In contrast, other studies report no relationship between self-reported stress levels and the degree of sleep bruxism [123–125]. Overall, although the majority of studies suggest that sleep bruxism is associated with stress, the specific stress-factors that correlate with sleep bruxism remain unclear.

### 3.2. Chewing Gum and Stress

People chew gum for a variety of reasons, including modulation of psychologic states, for example, to facilitate concentration, relieve stress, and reduce sleepiness. Many studies have examined the effects of gum chewing on stress.

#### 3.2.1. Chewing Gum and Stress Markers in Saliva

Analysis of stress markers in the saliva is a simple and useful method for determining stress levels in humans. In humans, gum chewing or bruxism-like activities under various stress conditions may influence the secretion volume of various stress markers in the saliva. Chewing leads to decreases in alpha-amylase activity (a sympathetic nervous system stress marker [126]), salivary cortisol levels (an endocrine system stress marker [28, 127]), and secretory immunoglobulin A (an immune system stress marker) [128, 129]. Bruxism-like activity during the presentation of a loud unpleasant sound prevents a stress-induced increase in salivary chromogranin A [130], a stress marker that reflects sympathetic activity [131]. Chewing and light teeth-clenching after stress loading lead to a rapid reduction of salivary cortisol levels [127]. Interestingly, a fast chewing rate [132] and a strong [133] chewing force induce a greater reduction in mental stress than a slow or weak force. Tasaka et al. [128] reported that chewing time affects the response of the endocrine system to mental stress, and continuous chewing for more than 10 min is effective for reducing stress, based on stress marker analysis in saliva. Contrary to these reports, however, chewing gum fails to attenuate salivary cortisol levels [33, 134]. The increase in the cortisol secretion is likely task-dependent. Also, these studies were performed at various times of day, and thus the conflicting results may be due to the diurnal alternations in cortisol secretion.

Pröschel and Raum [129] reported a positive association between chewing force and mean amplitude of the electromyogram of masticatory muscles. The mean electromyogram amplitude of the masticatory muscles during chewing increases with increased psychologic stress [135]. Psychosocial stress is associated with an increased chewing frequency and decreased appetite [136]. These findings suggest that chewing and bruxism-like activities are autonomic behaviors in response to stressful conditions, acting as stress-coping mechanisms. Niwa et al. [137] reported that chewing increases activity in the prefrontal cortex, which is involved in stress control, and leads to decreased stress markers in saliva [133].

#### 3.2.2. Chewing Gum and Experimental or Naturally Occurring Stress

Several studies have demonstrated the benefits of chewing on stress, since Hollingworth [138] reported that masticatory movement reduced excessive muscular tension and energy. Soon after the report by Hollingworth, however, foot-tapping was reported to produce the same relaxing effects, suggesting that the stress-reducing effects were not specific to gum chewing [139]. Therefore, the benefits of gum chewing on stress remain a matter of debate. The effects of gum chewing on naturally occurring stress are consistently reported to be beneficial. For example, Zibell and Madansky [28] investigated whether chewing gum affects perceived levels of everyday stress among subjects who regularly chew gum or among subjects who do not usually chew gum. Stress levels and stress-specific emotions, such as feeling anxiety or tension, decreased after chewing gum, indicating that gum chewing reduces levels of anxiety and stress. Smith [29] performed a cross-sectional study of occupational stress in full-time workers and found that non-gum-chewers complained significantly more often of stress at work and home compared with gum chewers, and gum chewers had a lower incidence of high blood pressure. An intervention study revealed that chewing gum reduces occupational stress both at and outside of work, reducing fatigue, anxiety, and depression and leading to a more positive mood [29]. Chewing gum is also associated with perceptions of better performance [140]. Further, chewing gum is associated in a linear dose-response manner with levels of perceived stress at work and home, as well as anxiety and depression [141]. Similar findings were reported for university students [142]. Erbay et al. [143] examined whether chewing gum is a useful addition to traditional medical treatment of patients with mild to moderate depression and indicated that while chewing gum is not directly effective for elevating a depressed mood, it may reduce the symptoms originating from depression.

On the other hand, the effects of chewing on stress are also variable in studies of experimentally induced stress. Scholey et al. [30] investigated the effects of chewing gum on multitasking efficiency. Chewing gum significantly increases self-rated levels of alertness, decreases self-rated levels of anxiety and stress, reduces salivary cortisol levels, and enhances overall task performance. The effects were the same regardless of the gum flavor. The authors [30] speculated that their findings are linked to the increased heart rate [144, 145] and increased cerebral blood flow [146] associated with chewing. Additional studies have reported a relationship between chewing under stress-inducing conditions and heart rate [147–151]. These findings suggest that an increase in cerebral blood flow [146, 152] and the related increase in glucose delivery [153] might act to reduce stress via an increase in the PFC glucose metabolism. Additionally, Kern et al. [154] demonstrated that an increase in glucose metabolism in the rostral mPFC is associated with a decrease in the salivary cortisol concentration following a stressful task. Numerous studies have reported an increase in cerebral activity after gum chewing [155–161] and demonstrated that the effect is specific to chewing gum and not just the chewing motion [162, 163]. Chewing gum ameliorates the effects of stress on mood, anxiety, and mental status [33, 134, 164]. One possibility is that the chewing-induced neural activation and psychologic and mental benefits improve task performance, which suppresses stress.

Notwithstanding the above reports, the effects of chewing gum on cognition and physiology are controversial. For example, the facilitative effects of chewing gum on memory [145, 153] have proved difficult to replicate [32], as has the accelerating effect of chewing gum on heart rate [32, 145]. The context-dependent memory effect demonstrated by Baker et al. [165] has not been replicated; thus the effects of chewing gum on context-dependent memory are conflicting [165–169]. In addition, Johnson et al. [33] detected no benefits of chewing on cortisol levels, state anxiety, or stress despite using a similar study design as Scholey et al. [30]. Torney et al. [34] also found no effect of chewing gum on mediating the level of stress experienced or on performance in a solvable anagram task. The anagram task used by Torney et al. [34] was only 5 min long, much shorter than the task used by Scholey et al. [30] (~20 min), suggesting that a greater period of chewing is needed to observe a reduction in stress [153].

Smith [134] examined whether gum chewing improves aspects of cognitive function and mood during exposure to a 75 dB stress-inducing noise. His findings revealed that chewing gum was associated with both more alertness and more positive mood. Reaction times were faster in subjects who were allowed to chew gum. Chewing gum also improved selective and sustained attention. Both heart rate and cortisol levels were higher during chewing, confirming that chewing gum has an alerting effect rather than a stress-reducing effect, consistent with another report [170]. Therefore, the findings regarding gum chewing are mixed, with some indicating that chewing gum is associated with significantly better alertness or vigilance [30, 33, 134, 148, 171, 172] and others indicating no benefit of chewing gum for attention, self-related alertness, and vigilance [173, 174]. The differences in these reports may derive from the duration of the study and time required for the task [174, 175].

Recently, the coping mechanism of chewing under noise stress conditions was examined using functional magnetic resonance imaging [31]. Gum chewing attenuated stress-induced activation of the bilateral superior temporal sulcus and left anterior insula [31]. Gum chewing reduced functional connections between the left anterior insula and the dorsal anterior cingulate cortex and inhibited the connectivity from the bilateral superior temporal sulcus to the left anterior insula [31]. Chewing gum under stress might act to attenuate the sensory processing of the stressor and inhibit the transmission of stress-related information in the brain [31].

## 4. Conclusions and Future Directions

Chewing or biting as a stress-coping behavior attenuates stress-induced diseases such as gastric ulcers and cognitive and psychologic impairments in rodents via suppression of stress-induced activation of the HPA axis and autonomic nervous reactions. The histaminergic nervous system may also be involved in the chewing-induced attenuation of stress-induced cognitive deficits. In humans, although the correlation between sleep bruxism and stress factors is controversial, many studies support an association between stress and sleep bruxism. Effects of chewing during stress are also conflicting. Gum chewing during stress may affect the levels of various stress markers in the saliva and plasma and increase attention, self-rated alertness, and vigilance.

Further studies are necessary to determine the possible causal relationship between sleep bruxism and stress factors. The amygdala and mPFC have a major role in stress-related behaviors and the mPFC also functions to regulate amygdala-mediated arousal in response to stress. Catecholamines such as 5-hydroxytryptamine dopamine and noradrenaline are involved in the corticolimbic circuitry, and gamma aminobutyric acid has a major role in amygdala functioning. Further studies focusing on the interactions between mastication and neuronal networks between the mPFC and amygdala and between the trigeminal nerve and cortical and limbic systems will help to clarify how mastication affects the expression of various stress-related markers. Studies using functional magnetic resonance imaging and functional near-infrared spectroscopy will be useful for analyzing brain activities in the mPFC and amygdala. More studies are necessary to clarify the benefits of gum chewing, by focusing on attention, alertness, vigilance, and others under task performance using functional magnetic resonance imaging and/or functional near-infrared spectroscopy in humans.
